# Aptamer-Based Carboxyl-Terminated Nanocrystalline Diamond Sensing Arrays for Adenosine Triphosphate Detection

**DOI:** 10.3390/s17071686

**Published:** 2017-07-21

**Authors:** Evi Suaebah, Takuro Naramura, Miho Myodo, Masataka Hasegawa, Shuichi Shoji, Jorge J. Buendia, Hiroshi Kawarada

**Affiliations:** 1 Department of Nanoscience and Nanoengineering, School of Advanced Science and Engineering, Waseda University, Tokyo 169-8555, Japan; t.naramura@akane.waseda.jp (T.N.); foreverfriends@ruri.waseda.jp or m.aq2116@gmail.com(M.M.); shojis@waseda.jp (S.S.); jorge.buendia@toki.waseda.jp (J.J.B.); kawarada@waseda.jp (H.K.); 2 Technology Research Association for Single Wall Carbon Nanotube (TASC), 1-1-1 Higashi, Tsukuba 305-8565, Japan; hasegawa.masataka@aist.go.jp; 3 Kagami Memorial Research Institute for Material Science and Technology, Shinjuku-ku, Tokyo 169-0051, Japan

**Keywords:** aptamer, adenosine triphosphate, nanocrystalline diamond, carboxyl termination

## Abstract

Here, we propose simple diamond functionalization by carboxyl termination for adenosine triphosphate (ATP) detection by an aptamer. The high-sensitivity label-free aptamer sensor for ATP detection was fabricated on nanocrystalline diamond (NCD). Carboxyl termination of the NCD surface by vacuum ultraviolet excimer laser and fluorine termination of the background region as a passivated layer were investigated by X-ray photoelectron spectroscopy. Single strand DNA (amide modification) was used as the supporting biomolecule to immobilize into the diamond surface via carboxyl termination and become a double strand with aptamer. ATP detection by aptamer was observed as a 66% fluorescence signal intensity decrease of the hybridization intensity signal. The sensor operation was also investigated by the field-effect characteristics. The shift of the drain current–drain voltage characteristics was used as the indicator for detection of ATP. From the field-effect characteristics, the shift of the drain current–drain voltage was observed in the negative direction. The negative charge direction shows that the aptamer is capable of detecting ATP. The ability of the sensor to detect ATP was investigated by fabricating a field-effect transistor on the modified NCD surface.

## 1. Introduction

Aptamers are single-stranded nucleic acids with selective binding ability obtained by the systematic evolution of ligands by exponential enrichment process from random sequences using an in vitro process [1]. Aptamers are ideal for biosensor applications because of their high affinity, specificity, sensitivity, and stability [2,3,4,5]. The selectivity and specificity of aptamers for a target are unique advantages over those of antibodies [6]. Detectable signals for adenosine triphosphate (ATP) detection can be easily generated by fluorescence techniques [7] and electrochemical methods [8]. Recently, diamond substrates have been recognized as good candidates for biosensing applications [9,10]. Compared with other candidates [11,12], diamond substrates are better electrochemical transducers because of their favorable chemical properties, outstanding biocompatibility [8,9,13,14,15,16], and large potential windows [15]. Nanocrystalline diamond (NCD) has high chemical stability and biocompatibility, and it is simple to functionalize [13,14] for various biological samples [10,17,18]. NCD produces good results for low level detection [17]. A label-free aptamer-based ATP detection system has been fabricated on NCD using fluorescence for detection [19]. Photolithography is one way to produce arrays on the NCD surface [20].

ATP is an important substrate in biological reactions and plays a key role in the bioenergetics of biological systems [21] and chemical compounds [22]. ATP binding aptamers were firstly introduced for oligonucleotide (DNA) applications and then extended to ribonucleic acid (RNA) applications [23,24]. ATP target aptamer detection has been extensively used for sensing [11] and medical research [7,25,26]. Label-free ATP detection by fluorescence has been achieved by transferring a labeled aptamer in the hybridized state spontaneously bound to ATP [27,28,29]. Adenosine is a component of many biological cofactors and combines with phosphate to form various chemical compounds [2].

Here, for ATP detection using dot pattern arrays, we report simple functionalization of the NCD surface by attaching biomolecules to the surface with a chemical linker [30]. We fabricated carboxyl-terminated NCD and developed a field-effect transistor (FET) device for quantitative ATP detection by an aptamer. NCD was terminated by carboxyl groups by dry treatment using vacuum ultraviolet (VUV) light and patterned by photolithography [19]. The supporting DNA immobilizes and hybridizes with the aptamer for ATP detection. Fluorine termination is used to lower the signal-to-noise ratio of the biosensor on the NCD surface [10,14,15,16,17,18,31]. Fluorine termination is better than oxygen or hydrogen as a passivation layer [32]. The resulting passivation effect shows a distinct contrast between the patterned region with partial carboxyl termination and the fluorinated background region. The resulting NCD with partial carboxyl termination is expected to be stable after denaturation. The FET sensor system was investigated by electrochemical measurements. The data show that ATP detection can be achieved using the threshold voltage shift signal.

## 2. Materials and Methods

To partially CH_3_ terminate the NCD surface, NCD on silicon [33] was exposed to methane-containing (3%) plasma (297 sccm hydrogen gas diluted with 3 sccm methane gas) for 30 min using a microwave plasma chemical vapor deposition system with a chamber pressure of 50 Torr, power of 1.2 kW, and temperature of ~700 °C. After 30 min, partial CH_3_ termination was achieved, and the flow of both gases was stopped, and the reaction system was cooled for 1 h.

Direct partial carboxyl termination was performed on the partially CH_3_-terminated NCD surface. NCD was functionalized by UV irradiation to directly produce carboxyl groups. UV irradiation was performed using a Xenon excimer lamp with a lamp power of 20 W to generate an ultraviolet laser with a wavelength of 172 nm. Pure oxygen was introduced into the chamber to achieve a pressure of 3 × 10^4^ Pa, and then UV irradiation was performed for 45 min at room temperature. X-ray photoelectron spectroscopy (XPS) was used to characterize the surface chemical change after carboxyl termination. XPS electron spectrometry was conducted using an Ulvac Φ 3300 (Ulvac-Phi, Kanagawa, Japan) with a monochromatic Al Kα X-ray source. 

**Figure 1:**
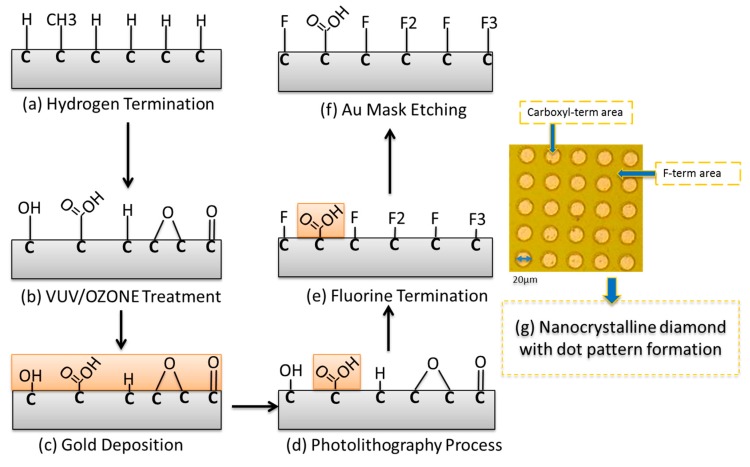
Schematic diagram of micropattern fabrication on the nanocrystalline diamond (NCD) surface. (**a**) Hydrogen termination; (**b**) Vacuum ultraviolet (VUV)/Ozone treatment; (**c**) Gold deposition; (**d**) Photolithography process; (**e**) Fluorine termination; (**f**) Au mask etching; (**g**) Optical microscopy image of the dot pattern formed on the NCD surface. The dots are terminated by carboxyl groups and regions outside the dots are fluorine-terminated as a background.

A micropattern was fabricated on the NCD surface by photolithography. Gold (150 nm thick) was deposited on the NCD surface. Before patterning by an aligner, the NCD surface was pre-baked for 20 min at 80 °C to remove all water molecules. The gold layer was coated with a resist film by spin coating, aged for 20 min at room temperature, and then baked at 100 °C for 5 min. The sample was then patterned with the aligner for 33 s. The negative pattern produced by photolithography was obtained by gold etching with KI/I_2_ etching solution. The outside of the dot pattern was exposed to C_3_F_8_ plasma (RIE-101iPH; Samco International Inc., Tokyo, Japan) for 15 s to generate a fluorine-terminated surface as a passivated layer outside the dot pattern. This process minimizes non-specific adsorption of supporting molecules and the aptamer. A schematic diagram of fabrication of the micropatterned NCD surface is shown in Figure 1.

Figure 1 shows a schematic diagram of NCD functionalization to produce the carboxyl-terminated surface. Micropatterning produced a dot pattern with 20 μm diameter dots separated by a distance of 20 μm. The dot patterns are partially carboxyl-terminated, while the fluorinated surface is the background. An optical microscopy image of the patterned NCD surface covered by gold before final etching with carboxyl- and fluorine-terminated regions is shown in Figure 1g.

Final etching was performed to remove the gold area on the NCD, and the remaining area covered with gold is carboxyl terminated. The supporting DNA was immobilized on the carboxyl-terminated dots after the final etching process. Carboxyl termination allows covalent immobilization of supporting DNA, which hybridizes with the aptamer for ATP detection. The resulting partially carboxyl-terminated NCD is expected to be stable after denaturation. In addition, fluorine termination allows non-specific adsorption, which lowers the signal-to-noise ratio and reduces non-specific bonding of the biosensor on the NCD surface.

**Figure 2:**
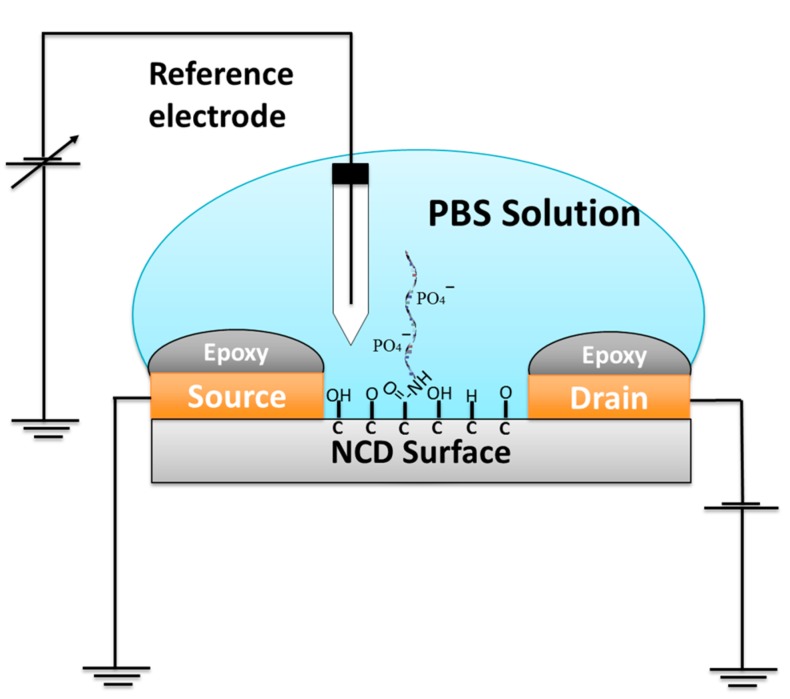
Schematic drawing of adenosine triphosphate (ATP) detection in solution gate field effect transistor (SGFET) based on a change in surface charge. PBS = Phosphate-buffered saline.

To develop an FET fabrication, NCD was deposited by a metal mask to create a source and a drain on the surface. The schematic drawing of FET is shown in Figure 2.

**Figure 3:**
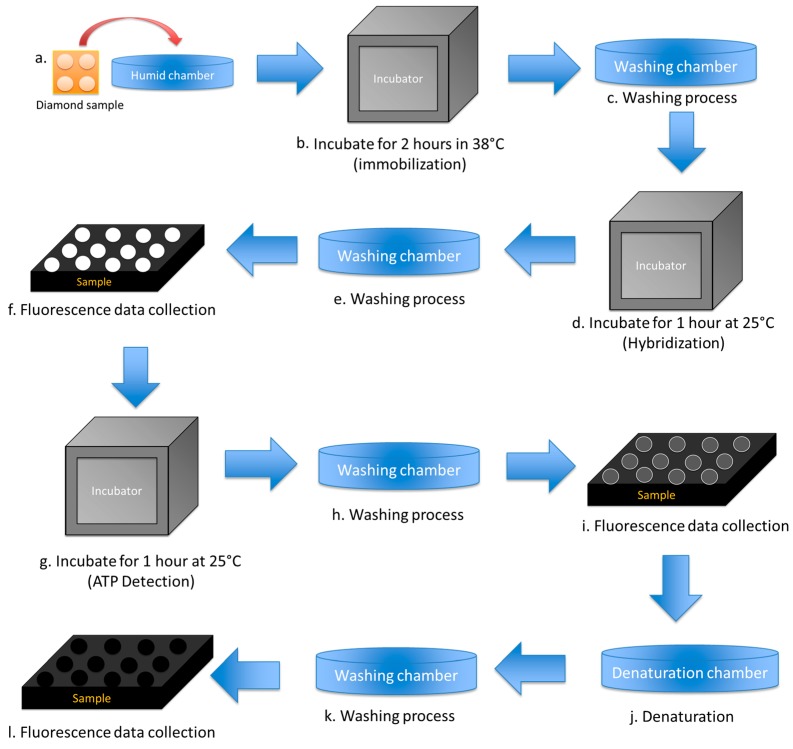
Experimental setup for data measurement. (**a**) Biomolecule dropped onto the surface and placed in the humid chamber; (**b**) Incubation for 2 h at 38 °C for the immobilization process; (**c**) Washing treatment; (**d**) Hybridization; (**e**) Washing treatment; (**f**) Fluorescence data collection for hybridization; (**g**) Incubate for 1 h at 25 °C for ATP detection; (**h**) Washing treatment; (**i**) Fluorescence data collection for ATP detection; (**j**) Denaturation treatment; (**k**) Washing treatment; (**l**) Fluorescence data collection for denaturation.

From Figure 2 schematic of NCD FET was fabricated as follows. Source and drain electrodes were deposited onto the NCD by a metal mask consisting of 150 nm of Au film covered by epoxy to protect it from the electrolyte solution. The channel was directly exposed to electrolyte solution with a 500 μm channel gate width [8]. *I–V* characteristics were measured by semiconductor analyzer (Keysight B150A, Tokyo, Japan). For fluorescence detection, signal measurement was conducted on the dot pattern region on the diamond surface; the detailed information is shown in Figure 3.

The experimental setup for biomolecule activation for fluorescence measurement is explained in Figure 3. All of the chemicals were provided by Sigma-Aldrich (Tokyo, Japan). For biomolecule activation, the carboxyl- and fluorine-terminated micropatterned NCD surface was activated in 0.1 M *N*-HydroxySuccinimide/0.4 M 1-ethyl-3-(3-dimethylaminopropyl) carbodiimide hydrochloride (1:1 volume ratio) for 1 h. The NCD surface was washed three times with deionized (DI) water and then dried before supporting-DNA immobilization. Washing was performed because it improves the immobilization capability [34]. The sequence of the supporting DNA complementary to the DNA aptamer is the 21-mer NH_2_-5′-CCACGGAC-TACTTCAAAACTA-3′ and the fluorescently labeled DNA aptamer has the sequence 3′-GGTGCCTG-ATGAAGTTTTGAT-5′-Cy5. The supporting amine-modified DNA was diluted three times with saline–sodium citrate (SSC) to give a final concentration of 20 μM. A small drop of the supporting solution was manually deposited on the diamond surface, covered with a plastic slip, and incubated at 38 °C for 2 h in a humid chamber (step b). The sample was washed once with wash buffer (1 M phosphate buffer solution and 0.1% Tween-20) for 5 min and then three times with DI water for 5 min before drying under an air flow (step c). The supporting DNA plays an important role in the hybridization process with the aptamer. Here, we used a single-stranded oligonucleotide that can be repeatedly used for hybridization and ATP detection.

After the supporting DNA was immobilized on the carboxyl-terminated diamond surface, the aptamer bound to Cy-5 was diluted two times with SSC to give a final concentration of 10 μM and then directly dropped onto the diamond surface. The aptamer was hybridized with the supporting DNA at 25 °C for 1 h in a humid chamber (step d, Figure 2). The hybridized surface was rinsed once with Tris-ethylenediaminetetraacetic acid (TE) buffer for 5 min and then three times with DI water for 5 min (step e). Hybridization was observed by an epifluorescence microscope (Olympus IX71; Olympus, Tokyo, Japan) as a fluorescent spot. The epifluorescence microscope captured the fluorescence intensity of the dot pattern area. The fluorescence captures the data through a recorded image, where the image shows the dot pattern as a high contrast and the background area as a low contrast (step f). Every image collected about 1800 dots and covered the 90 × 90 dot pattern. The fluorescence signal is due to the reaction between the carboxyl compound and the biomolecule on the diamond surface. 

ATP was introduced to double-stranded DNA (0.1 mM) and incubated at 25 °C for 1 h (step g, Figure 2). In the ATP detection process, the diamond surface was rinsed once with TE buffer for 5 min and then three times with DI water for 5 min (step h). Therefore, ATP is detected as a decrease in the fluorescence and the dot pattern becomes darker than in the previous image and approaches the intensity of the background. This method has the advantage of avoiding physical adsorption of ATP on the substrate. Denaturation was performed in urea (8.3 M) for 30 min (step j) and the surface was then rinsed with DI water three times for 5 min each time (step k). In the denaturation process, the aptamer/ATP complex is removed from the substrate surface. The diamond surface still contains the supporting DNA, but without the aptamer and other molecules.

## 3. Results and Discussion

### 3.1. X-ray Photoelectron Spectroscopy Analysis

Quantitative analysis by X-ray photoelectron spectroscopy (XPS) is very important to investigate the substrate surface and monitor the effectiveness of the surface reaction with VUV treatment. XPS experiments were performed to determine the amount of carboxyl groups that formed on the diamond surface by VUV irradiation. Carboxyl groups successfully formed on the diamond surface with a coverage of above 1%. The coverage of carboxyl groups as a linker between supporting DNA and the diamond surface is an important factor in the immobilization process. The surface density of carboxyl groups determines the effective number of probe DNA molecules that can immobilize on the diamond surface.

**Figure 4:**
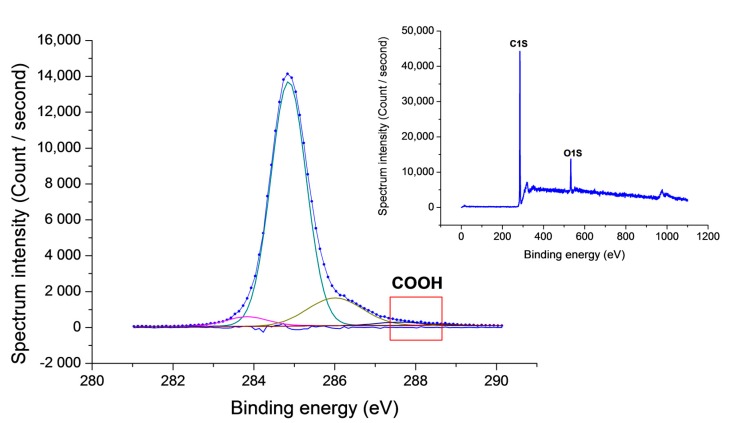
X-ray photoelectron spectroscopy (XPS) data of the oxidized diamond surface following VUV irradiation for 45 min. The insert shows the wide graph for the carboxyl coverage.

Figure 4 shows the carbon and oxygen coverage of the diamond surface after oxygen termination. Table 1 summarizes the XPS results for each type of chemical bonding state after VUV irradiation for 45 min. The sp^2^ peak of C 1s from diamond is detected at its typical position of 283.93 eV. The peaks at 284.85, 285.86, and 286.57 eV correspond to C–C, C–O, and C=O groups, respectively. The peak from carboxyl groups is at 288.12 eV. By hydrogen termination, the possible surface functional groups are CH, CH_2_, and CH_3_. Both CH_2_ and CH_3_ groups can be oxidized to hydroxyl groups. Changing the bonding configuration of the CH_3_ group structure can introduce diverse types of oxygen-related components to the diamond surface. The CH_3_ terminals at the step edge of the diamond surface can be oxidized to give a high coverage of carboxyl acid groups [35]. Carbon atoms with three dangling bonds are required to obtain COOH groups. With strong oxidation by the VUV excimer laser, CH_3_ becomes COOH. A high density of ozone and excited oxygen are required in the irradiation process to change CH_3_ to COOH [34,36]. By VUV treatment, the average thickness of O is 0.6 monolayers. This means that the diamond surface is uniformly covered by O. Considering one monolayer of diamond, the maximum oxygen coverage by oxygen groups estimated from the XPS data is about 60%. From that value, as much as 60% will be immobilized with supporting DNA, and aptamer hybridization depends on the density and concentration of the supporting DNA.

### 3.2. Fluorescence Signal Detection

**Figure 5:**
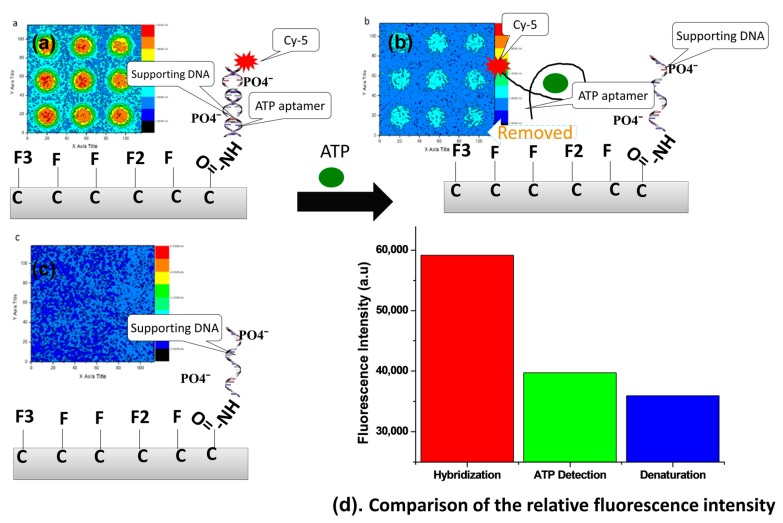
Fluorescence signals at different steps of the sensor operation. (**a**) Hybridization of the supporting DNA with the aptamer modified with Cy-5 as a fluorescent indicator; (**b**) ATP detection via the aptamer; (**c**) Denaturation, which occurred when ATP and the aptamer were removed from the sensor and the DNA became single stranded; (**d**) Comparison of the relative fluorescence intensities at the three observation points for similar areas.

Fluorescence signal detection was performed after hybridization, ATP detection, and denaturation. These three steps are shown in Figure 5. Aptamer binding to ATP was used to examine ATP detection by the characteristic fluorescence signal of Cy-5 with the change of intensity method. When the supporting DNA was immobilized on the diamond surface, the epifluorescence microscope did not capture a fluorescence signal inside or outside the dot pattern. Supporting DNA molecules were immobilized on the diamond surface without a fluorophore label, so it is not surprising that no signal was detected by the microscope in this case. When the aptamer modified with Cy-5 as a fluorophore was hybridized with supporting DNA, a fluorescence signal was detected. Thus, hybridization between the supporting DNA and the aptamer occurred. The fluorescence signal was only detected inside the dot pattern, and the background area was dark. When ATP was introduced onto the NCD surface, the aptamer was released from the supporting DNA and bound to ATP. The specific molecule can be detected by an aptamer which has very specific bonding. As a result, the fluorescence signal drastically weakened. The decrease of the fluorescence is considered to be detection of ATP. The fluorescence signal completely disappeared with denaturation of the remaining aptamer hybridized with supporting DNA. Hybridization means that the supporting DNA immobilized by the carboxyl groups on the diamond surface hybridized with the aptamer. The indicator of successful hybridization is an increase of the fluorescence signal intensity inside the dot pattern. Coupling of the supporting DNA with the aptamer is considered to be a biosensor. Figure 5 shows the intensity of the fluorescence signal when hybridization occurred on the diamond surface.

In the three different steps, the fluorescence signal intensity change is important to determine the surface functionalization and initialization of the biosensor. The appearance of a fluorescence signal is used as an indicator of the biomolecule activity. A fluorescence signal was produced with an oxygen monolayer coverage of 60%, which provides sufficient DNA immobilization sites (see Figure 5a). Hybridization between the supporting DNA and the aptamer to spontaneously form double-stranded DNA was also confirmed by fluorescence measurements. The fluorescence intensity linearly increased when the aptamer was coupled with the supporting DNA. From Figure 5b, when ATP molecules were introduced into the double-stranded DNA (incubation time 1 h), the aptamer was released from the supporting DNA and formed a complex with ATP. Thus, the ATP molecules removed the aptamer from the supporting DNA, which became single stranded. The balanced energy of the double-stranded DNA becomes disturbed by ATP on the diamond surface if the total binding energy of the double-stranded DNA is lower than that of ATP and its aptamer, then, the aptamer will be released from the complementary DNA to bind with ATP [37]. The fluorescence dot pattern on the diamond surface disappeared as the aptamer was removed by ATP. That is, the intensity of the fluorescence signal immediately decreased as the aptamer was removed from the supporting DNA. The intensity decrease depended on how much ATP was detected by the aptamer. Thus, the decrease of the intensity of the fluorescence signal was used for ATP detection. The aptamer plays an important role because it conjugates with ATP. This process was confirmed by the decrease of the fluorescence intensity upon ATP addition. The accuracy of ATP detection was dependent on covalent bonding with biomolecules on the NCD surface. Carboxyl termination within the dots and fluorine termination outside the dots contributed to minimizing non-specific adsorption of DNA and ATP because of the repulsive force between negatively charged atoms from the NCD surface and the molecules. Figure 5d compares the relative intensities of the fluorescence in the three steps. The highest intensity is observed after the hybridization process of DNA modified with Cy-5 on the sensor surface. After ATP was introduced, the fluorescence intensity decreased to around 56% of the hybridization intensity. This fluorescence signal decrease is an indicator of ATP detection by the aptamer. The aptamer was removed from the supporting DNA by ATP, decreasing the amount of the double-stranded supporting DNA–aptamer complex. To convert all of the double-stranded DNA to single strands, denaturation was performed to restore the immobilization conditions without any aptamer bound to the supporting DNA. That is, only supporting DNA remained on the diamond surface after denaturation. The fluorescence intensity after denaturation was similar to the background intensity, and it was about 67% lower than the hybridization signal.

**Figure 6:**
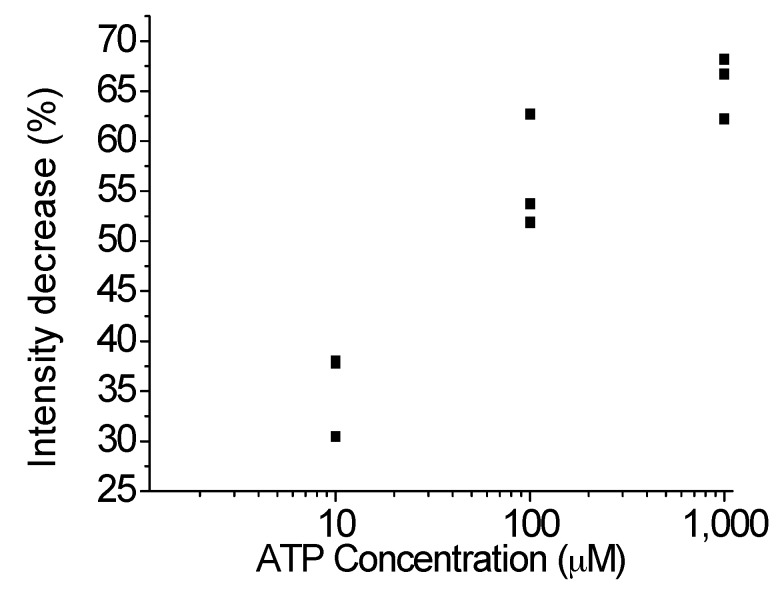
Relationship between the fluorescence intensity for different ATP concentrations. Every single data point is the average of three measurements.

Figure 6 shows the relationship between the ATP concentration and the fluorescence intensity decrease. A low ATP concentration results in a low fluorescence intensity decrease. When a high concentration of ATP is added, more aptamer will be detected by ATP and released from the supporting DNA. In contrast, if a small concentration of ATP is added, a small amount of aptamer will be detected by the aptamer and released from the supporting DNA. This shows that the amount of ATP is proportional to the amount of aptamer released from the supporting DNA. The lower limit is 10 μM ATP and the upper limit is 1 mM ATP.

### 3.3. Threshold Voltage Shift for Carboxyl Termination

We fabricated a FET containing the carboxyl-terminated NCD. The threshold voltage shift after each step of the treatment was determined by determining the FET current–voltage (*I*–*V*) characteristics. To understand the charge carrier mechanism corresponding to the FET characteristics, hybridization and ATP detection were investigated with changing current.

**Figure 7:**
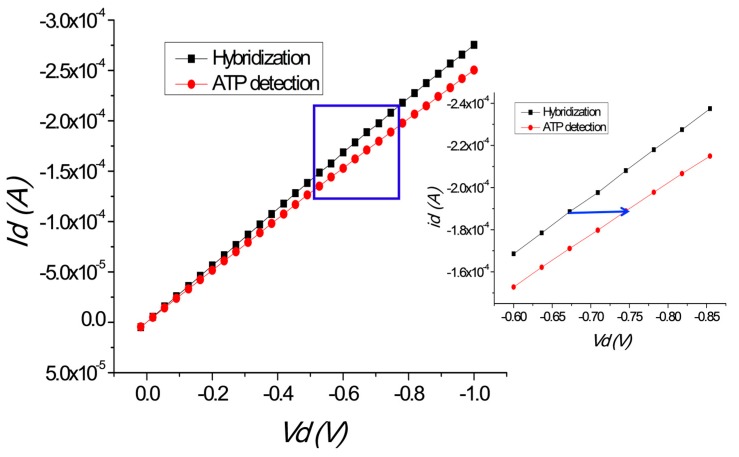
*I*–*V* (drain current *I_d_* and drain–source voltage *V_d_*) characteristics of the modified diamond-based field-effect transistor (FET) showing ATP activity (gate voltage −0.2 V).

Figure 7 shows the *I*–*V* characteristics. The applied gate voltage was −0.2 V and we performed hybridization and ATP detection. First, *dV/I_d_* increases because of the negative charge of the NCD surface originating from the supporting DNA backbone. When the supporting DNA becomes double stranded by coupling with the aptamer, the negative charge in the channel area doubles and the current at the gate area increases [35,38]. The negative charge of the channel depends on the negative charge originating from the DNA backbone. Holes will rise to the surface when they are attracted by the negative charge near the surface. This will result in a current change. The current will increase as the flow of moving holes increases. When ATP is added and forms a complex with the aptamer, the negative charge on the diamond surface decreases with changing current. The current decrease depends on removal of the negative charge from the diamond surface as the aptamer is lost during ATP detection. Holes from the surface appear as negative charge is removed from the channel area. This results in a current decrease, which indicates ATP detection. Figure 6 shows that the current decreases with ATP detection. The current from the hybridized sample is higher than that during ATP detection. The voltage shift from hybridization to ATP detection is 7.28 mV in the negative direction at a source drain current of 18.8 mA. This means that the charge distribution from the aptamer backbone changes because negative charge is distributed in the channel layer. The charge distribution induces electrons, which are attracted to the positive holes on the diamond surface. The number of holes depends on the size of the accumulation layer. The hole concentration decreases when ATP attaches to the aptamer and is removed from the layer.

### 3.4. Reusability of the Aptasensor

**Figure 8:**
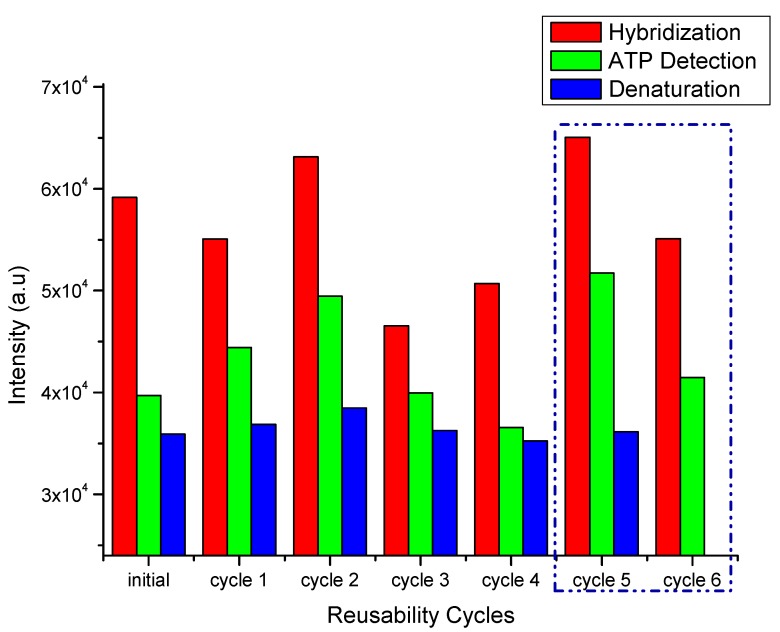
Reusability of the aptasensor for seven cycles over two weeks.

The sensor showed good reproducibility, which is attributed to the fact that the aptamer covalently immobilized on the carboxyl-modified diamond surface was stable and maintained its ability to selectively bind to ATP. The stability of the diamond sensor was investigated by reusing the sensor. Hybridization, ATP detection, and denaturation were repeated multiple times to determine the characteristics of ATP detection and hybridization. The fluorescence signals indicated that the characteristic hybridization process stably occurred. The fluorescence intensity after the hybridization process was almost stable ((50 − 60) × 10^3^). The fluorescence intensity from the sensor at each step during seven cycles is shown in Figure 8.

**Figure 9:**
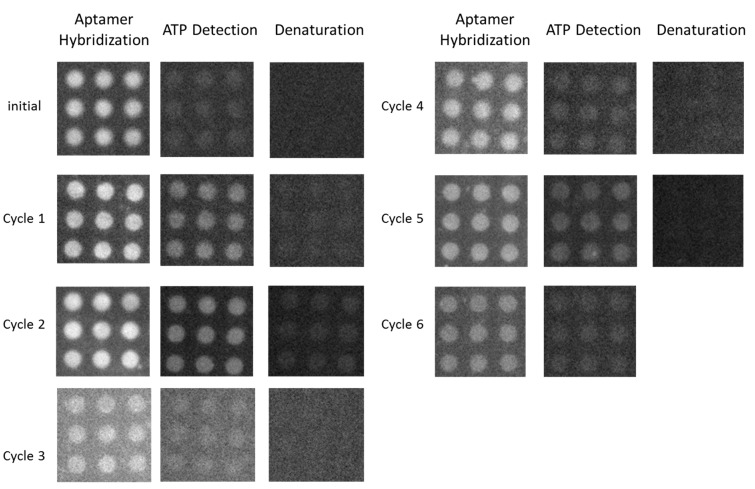
Fluorescence intensity variation during aptasensor reuse over two weeks.

ATP detection was repeated seven times over two weeks. The initial data are for the first time the sensor was used. Cycle 1 was performed 11 days after the sensor was left in a refrigerated humid chamber. The cycle 1 data are acceptable. Cycle 2 was performed on the same day as cycle 1. Cycles 3 and 4 were performed on day 12, and cycles 5 and 6 were performed on day 13. Investigation of the reusability and long-term storage of the sensor were combined for one sample. The reusability data give information about the strength of the bonds between the carboxyl groups and the supporting DNA, and the long-term storage indicates the stability of the sample. These two factors are closely related. From the results, it is a good sensor because it remains stable for a long time, and the sensor still functions well when used several times. The maximum fluorescence intensity is observed after DNA hybridizes with the aptamer, and it decrease after ATP-driven release of the aptamer. In contrast, amine termination by a nitrogen/hydrogen radical beam only provides reusability for a short time [6], which is good for nitrogen vacancy center fabrication [39]. The carboxyl-modified sensor is a good candidate for frequent reuse. In the experiments, an ATP concentration of 1 mM was used for cycles 2–4, while ATP concentrations of 10 and 100 μM were used for cycles 5 and 6, respectively. The sensor retained its activity after several cycles. Figure 8 confirms the reusability of the aptasensor, where the aptamer coupled with the supporting DNA. Figure 9 shows the fluorescence intensity change of the diamond surface sensor for multiple cycles.

Figure 9 shows the change of the fluorescence intensity when the aptasensor was used seven times. The stability of the sensor is indicated by the fluorescence signal in the hybridization step. The fluorescence signal following hybridization is stable after reusing the aptasensor seven times. The dot pattern remains on the diamond surface after seven cycles, indicating detection by the aptamer is still possible. This confirmed that the NCD-based aptasensor is reusable.

## 4. Conclusions

DNA immobilization has been achieved by carboxyl modification of the NCD surface. Detection of ATP by aptamer binding with the carboxyl-modified diamond surface was successfully achieved. Optical detection of ATP was performed using a fluorescent dye and determined from the voltage shift of the current–voltage characteristics. Above 1% carboxyl coverage provides sufficient biomolecule bonding for epifluorescence detection. Low coverage of carboxyl can perform properly for sensing applications, especially for ATP detection. The aptamer can detect ATP on the diamond surface at low concentration (10 μM), and the highest concentration considered was 1 mM. From the FET characteristics, the voltage shift from hybridization to ATP detection is 7.28 mV in negative charge, which means that the charge distribution on the diamond layer is from the backbone charge of the supporting DNA.

## Figures and Tables

**Table 1:** Core-level C 1s binding energies of various types of chemical groups on the diamond surface after VUV irradiation for 45 min.

Chem. State	Position (eV)	Height (C in S)	Area (Gaussian %)	% Area
Sp2	283.93	1154	1928	10.3
C-C	284.85	14607	14730	78.7
C-O	285.86	1411	1108	5.9
C=O	286.57	529	738	3.9
COOH	288.12	233	214	1.1
